# Infection of the Stable Fly, *Stomoxys calcitrans*, L. 1758 (Diptera: Muscidae) by the Entomopathogenic Fungi *Metarhizium anisopliae* (Hypocreales: Clavicipitaceae) Negatively Affects Its Survival, Feeding Propensity, Fecundity, Fertility, and Fitness Parameters

**DOI:** 10.3389/ffunb.2021.637817

**Published:** 2021-02-24

**Authors:** Steve B. S. Baleba, Ayaovi Agbessenou, Merid N. Getahun, Komivi S. Akutse, Sevgan Subramanian, Daniel Masiga

**Affiliations:** International Centre of Insect Physiology and Ecology (icipe), Nairobi, Kenya

**Keywords:** *Metarhizium anisopliae*, *Stomoxys calcitrans*, biological parameters, pre-lethal effects, ICIPE 30

## Abstract

Entomopathogenic fungi can cause substantial mortality in harmful insects. Before killing the insect, these pathogens start by negatively affecting the biological parameters of the host. Prior to our study, the information about how fungal exposure affects the biological parameters of the stable fly, *Stomoxys calcitrans* was still elusive. Therefore, we aimed to assess the infection of *S. calcitrans* with some *Metarhizium anisopliae* strains, and their impact on feeding, fecundity, fertility and other life-history traits of this fly. Among the 11 *M. anisopliae* strains screened, we identified ICIPE 30 as the most virulent strain against *S. calcitrans*. We observed that the infectivity of this strain was sex and age-dependent. Infected male *S. calcitrans* died earlier than their counterpart females. Older infected *S. calcitrans* died faster than infected young ones. Also, male and female *S. calcitrans* successfully transmitted ICIPE 30 conidia to their mates. We demonstrated that infection by ICIPE 30 extended the feeding time of *S. calcitrans* and consequently reduced the feeding probability of the fly and the amount of blood taken. Using a dual test oviposition bioassay, we determined that uninfected gravid female *S. calcitrans* avoided laying eggs on substrates amended with ICIPE 30 conidia. We showed that these conidia could lower the hatchability of the eggs deposited by gravid females. Using, a no-choice test, we showed that gravid female *S. calcitrans* infected with ICIPE 30 laid fewer eggs than uninfected females and those eggs hatched less. Using 11 strains of *M. anisopliae* and four high concentrations of ICIPE 30 conidia, we verified that *S. calcitrans* larvae were not susceptible to fungal infection. Further, we showed that though these larvae were tolerant to fungal infection, there was a significant effect on their fitness, with contaminated larvae having a small bodyweight coupled with longer developmental time as compared to uncontaminated larvae. Our study provides detailed information on how fungal infection affects the biology of *S. calcitrans* and the potential of using *M. anisopliae* ICIPE 30 as a biopesticide to reduce the fly population. Such knowledge can assist in developing fungal-based control strategies against this harmful fly.

## Introduction

*Metarhizium anisopliae* (Hypocreales: Clavicipitaceae) is a ubiquitous entomopathogenic fungus infecting a wide range of insect hosts and used for biological control (Brunner-Mendoza et al., 2019). Its mode of action involves attachment to the host's cuticle, germination, epicuticle penetration and dissemination inside the insect body as hyphae (Ortiz-Urquiza and Keyhani, 2013). This infection mechanism is facilitated by a group of enzymes including hydrolases, proteases, chitinases and lipases (Brunner-Mendoza et al., 2019). When proliferating inside insect tissues, blastospores produce toxic molecules (e.g., destruxins) that induce pathogenesis, paralysis, cellular alterations and dysfunction of the middle intestine, malpighian tubules, and muscle tissues (Samuels et al., 1988). These cascades of events ultimately provoke insect death 3–7 days after infection (Mondal et al., 2016). It has been demonstrated that the speed with which an insect succumbs from fungal infection could depend on the fungal strain (Valero-Jiménez et al., 2014), insect species, insect sex, and age (Maniania and Odulaja, 1998). However, before the death occurs, several pre-lethal reactions including the reduction in development, feeding propensity, and reproduction can be observed in infected insects.

Several laboratory and field trials have demonstrated the lethal and pre-lethal effects of *M. anisopliae* infection in insects. In blood-feeding insects, this fungus is known to cause high mortality in immature and adult stages. For instance, *M. anisopliae* has proven to reduce survival of different larval stages of *Aedes aegypti, Anopheles stephensi*, and *Culex quinquefasciatus* (Diptera: Culicidae) (Greenfield et al., 2015; Ravindran et al., 2016). In *Phlebotomus papatasi* (Diptera: Psychodidae), *M. anisopliae* reduces adult emergence when applied to larval food (Zayed et al., 2013; Alkhaibari et al., 2017). Adults of the tsetse fly, *Glossina morsitans* (Diptera: Glossinidae) that emerge from *M. anisopliae*-infected pupae suffer from high mortality (Kaaya and Munyinyi, 1995). Maniania (2002) found that in the field *M. anisopliae* can reduce the population of *Glossina* spp. by 82.4%. Laboratory-based bioassay revealed high mortality in *Anopheles gambiae* and *C. quinquefasciatus* owing to *M. anisopliae* infection (Scholte et al., 2003). In *Meccus pallidipennis* (Hemiptera: Reduviidae), a vector of *Trypanosoma cruzi*, Flores-Villegas et al. (2016) observed that individuals treated with *M. anisopliae* died sooner than untreated individuals. In addition to this lethal effect, infection by *M. anisopliae* is also known to induce pre-lethal effects on hematophagous insects. This has been shown in *An. gambiae* (Scholte et al., 2003) and *Ae. aegyptis* (Paula et al., 2011); where a reduction of feeding and reproduction was reported after exposure to *M. anisopliae*. Here, we studied the infection of *M. anisopliae* in the stable fly, *Stomoxys calcitrans* (Diptera: Muscidae) and its consequences on the feeding, fecundity, fertility, and life-history traits of this fly.

*S. calcitrans* is a cosmopolitan blood-feeding dipteran involved in the mechanical transmission of viruses (e.g., West Nile fever virus, Rift Valley fever virus), bacteria (e.g., *Bacillus anthracis, Pasteurella multocida*), protozoa (e.g., *Trypanosoma evansi, Besnoitia besnoit*), and helminths (e.g., *Habronema microstoma, Dirofilaria repens*) in various hosts including cattle, camels, horses, dogs, and humans (Baldacchino et al., 2013). During its high infestation periods, *S. calcitrans* can induce a reduction of 40–60% in milk yield and 19% in cattle weight gain (Walker, 1990; Carn, 1996) as a result of the nuisance caused to livestock. In cattle industries, losses attributed to *S. calcitrans* are estimated to be around $2.2 billion annually (Taylor et al., 2012). As all holometabolous insects, the development of *S. calcitrans* goes through an egg, three larval instars (with the size and morphology varying across the three instars (see Friesen et al., 2015), pupae and adult stages. The development of *S. calcitrans* occurs in herbivorous dung (Baleba et al., 2019) and rotting organic matter such as silage, hay, grass clippings, and garden compost (Cook et al., 2018).

The use of the entomopathogenic fungus *M. anisopliae* as a control agent against *S. calcitrans* has already been investigated in various studies; with results showing the high susceptibility of adults, but not larvae (Moraes et al., 2008). The *M. anisopliae* strain Ma135 was reported to kill more than 90% of *S. calcitrans* adults (López-Sánchez et al., 2012). When aspersed on dairy cattle, Cruz-Vazquez et al. (2015) established that the *M. anisopliae* strain Ma134 reduce populations of S. calcitrans by 73%. To our knowledge, there are no studies in the literature addressing the pre-lethal effect of *M. anisopliae* infection in *S. calcitrans* or the influence of the age or sex of the fly. Moreover, there is no evidence of the horizontal transmission of *M. anisopliae* conidia in *S. calcitrans*. Thus, our main aim was to study the lethal and pre-lethal effect of *M. anisopliae* infection in *S. calcitrans*. Specifically, we tested whether: (1) infection of *S. calcitrans* by *M. anisopliae* conidia would be sex- and age-dependent; (2) *M. anisopliae* conidia would be horizontally transmitted in *S. calcitrans*; (3) *M. anisopliae* infection would negatively impact the feeding propensity, fecundity and fertility of *S. calcitrans*; (4) there would be a trade-off between the tolerance of *S. calcitrans* larvae to *M. anisopliae* infection and their larval life-history traits.

## Materials and Methods

### *M. anisopliae* Strain Culture

The eleven strains of *M. anisopliae* used in our experiment were obtained from the *icipe*'s Arthropod Germplasm Center (Mweke et al., 2018; Akutse et al., 2020). The strains were cultured on Sabouraud Dextrose Agar (SDA) medium using 90-mm Petri dishes and maintained in the darkness at 25°C. Two weeks after the start of the culture, we harvested conidia of each strain by scraping the surface of the sporulated cultures using a sterile spatula. We suspended conidia of the different strains in 10 ml of distilled water with 0.05% Triton X-100 in universal bottles containing 3–5 glass beads (3 mm in diameter per bottle) each. The mixture was then vortexed for 5 min at 700 rpm to homogenize the suspension. Using an improved Neubauer haemocytometer under the light microscope, we determined the conidia concentration of each strain suspension following the protocol described by Lacey (2012). Before each bioassay, we tested the ability of conidia to germinate by spreading 100 μl of each strain suspension (titrated at 3 × 10^6^ conidia ml^−1^) on SDA plate. We sealed the inoculated plates with Parafilm membrane and incubated them in complete darkness at 25°C. At 18 h post-incubation, we flooded the plates with lactophenol aniline cotton blue to stop the germination process and stain the spore to ease their visibility for counting. Following this, we determined the number of conidia that germinated by counting 100 randomly selected conidia beneath each coverslip under a light microscope (400×). Conidium was considered as germinated if the length of its germ-tube was at least twice its diameter (Lacey, 2012). For each strain, we used five plates as replicates.

### *S. calcitrans* Colony

Individuals of *S. calcitrans* used in all our experiment were obtained from the “International Center of Insect Physiology and Ecology (*icipe*)” Animal Rearing and Quarantine Unit (ARCU) in Nairobi, Kenya (1° 13′ 12″ S, 36° 52′ 48″ E; ≈ 1,600 m above sea level) colony. This colony was established and maintained as described in Baleba et al. (2019). Briefly, wild individuals of *S. calcitrans* were captured at *icipe* campus using Vavoua traps (Laveissière and Grebaut, 1990), maintained inside a cage (75 cm × 60 cm × 45 cm) and fed twice per day (800 and 1,600 h) on defibrinated bovine blood poured on moistened cotton to initiate reproduction. Once gravid females were obtained, we exposed them to rabbit dung (fermented in a plastic bag for 1 week) placed in plastic containers (21.5 cm × 14.5 cm × 7.4 cm) for oviposition. After 24 h, we transferred the exposed containers to another cage (75 cm × 60 cm × 45 cm), and we monitored the development of the larval and pupal stages until adult emergence. We fed emerged adults with bovine blood and repeated the previously described above. We reared all the insects and performed our experiments in a laboratory under buffered conditions of 25 ± 5°C 65 ± 5% relative humidity, and 12L:12D photoperiod.

### Effect of *M. anisopliae* Infection on the Survival of *S. calcitrans* Adults

We determined the pathogenicity and virulence of 11 strains of *M. anisopliae* on *S. calcitrans* adults following the contamination protocol used by Wamiti et al. (2018) on *Glossina fuscipes fuscipes* (Diptera*:* Glossinidae). In this protocol, the contamination device (Supplementary Figure 1A) is comprised of a cylindrical plastic tube (95 mm × 48 mm) which has an inner part covered by a velvet carpet material impregnated with fungal dried conidia. Adult flies were gently introduced into the contamination device and allowed to pick conidia. After this period of exposure, flies were gently removed, and transferred in another cylindrical plastic tube free of conidia. In all our experiment, we used 0.1 g of conidia evenly spread on the velvet carpet, and exposed flies to conidia for 10 min. After transferring the fungus-exposed flies in a clean cylindrical plastic tube, we provided them with blood and recorded the number of dead flies daily for 7 days. We removed cadavers found inside the plastic tube using sterilized forceps and incubated them in Petri dishes containing moistened filter paper to assess the outgrowth of the applied fungal conidia (Supplementary Figure 1B). We used fungus-free flies as control. For each treatment, we used 10 flies and replicated the experiment five times.

### Effect of Sex and Age on *M. anisopliae* ICIPE 30 Infectivity in *S. calcitrans*

We used newly emerged (24 h old) males and females [differentiated based on the size of the two compound eyes that are smaller and more widely separated in females (dioptic) than in males (holoptic)] to see whether the pathogenicity of *M. anisopliae* varied between the sex of *S. calcitrans*. In the earlier experiment, we identified ICIPE 30 as the most virulent *M. anisopliae* strain against *S. calcitrans* (see results section). Here, we used this strain (0.1 g of dried conidia) to contaminate 10 males and 10 females following the protocol previously described above (Wamiti et al., 2018). As a control, we used unexposed males and females. For the age effect bioassay, we used only 10 exposed female flies (to account for any bias resulting from sex effect) of 1, 7, and 14 days old. The control groups consisted of fungus-free flies of 1, 7, and 14 days old. In both bioassays, we provided each group with bovine blood and recorded individual mortalities daily for 7 days. To confirm whether the death of the flies was caused by *M. anispoliae* ICIPE 30 infection, we placed dead flies in Petri dishes (9 cm) containing moistened filter paper to initiate fungal sporulation on the cadaver surfaces. We replicated each experiment five times.

### Horizontal Transmission of *M. anisopliae* ICIPE 30 Conidia by *S. calcitrans*

Before testing whether *M. anisopliae* ICIPE 30-exposed males and females could transmit conidia to their conspecific mates, we aimed to determine whether the number of conidia carried by *S. calcitrans* individuals could vary between sex and across time. To do so, we chilled 5 males and 5 females (2 days old) in ice for 2–3 min to induce a coma. Using fine sterilized forceps, we gently placed the immobilized flies inside the cylindrical plastic tube (on top of velvet carpet containing 0.1 g of ICIPE 30). After recovered from the coma, we allowed the flies to walk on conidia for 30 min, then individually introduced them inside universal bottles containing 2–5 glass beads and 1 ml of sterile distilled water with 0.05% Triton X-100. The bottles with the exposed flies were thereafter vortexed for 5 min (to remove conidia from the insect's body) and estimated the number of conidia carried by each individual using the Neubauer haemocytometer. To assess how the number of conidia carried by each individual be across time, we transferred the exposed male and female flies in a cleaned cage (15 cm × 15 cm × 20 cm), waited for 2, 4, 6, and 8 h before proceeding with the conidia quantification as previously described.

With a slight modification, we followed the protocol described by Maniania et al. (2013) to perform the horizontal transmission assay. We contaminated 5 males (donors) with 0.1 g of *M. anisopliae* ICIPE 30 conidia for 10 min then transferred them into another clean cage (15 cm × 15 cm × 20 cm). Four hours after this process, we transferred these males inside a clean cylindrical plastic tube and paired them with 5 fungus-free females (receivers). We use the same protocol to pair fungus-exposed females (donors) with fungus-free males (receivers). We considered fungus-free males and females as control. In all the treatments, we provided our flies with blood and recorded their mortality daily for 7 days. To confirm whether the dead in both sexes was induced by *M. anisopliae* infection, we placed separately the dead bodies of male flies in Petri dishes (9 cm) containing moistened filter paper to later assess fungal growth on the cadaver surfaces. We used five replicates in all the bioassays.

### Impact of *M. anisopliae* ICIPE 30 Infection on the Feeding Propensity of *S. calcitrans*

Here, using the previous contamination device, we exposed female *S. calcitrans* (2 days old) with the *M. anisopliae* strain ICIPE 30. To determine the effect of this fungal infection on the feeding propensity of *S. calcitrans*, we recorded three parameters, namely (1) the feeding duration, (2) the proportion of blood-fed, and (3) the amount of blood consumed. We determined the feeding duration by recording the time taken by an individual fly to get engorged after inserting its proboscis into the blood source (Supplementary Figure 2B). The proportion of blood-fed corresponded to the number of flies (in a group of 10 individuals) that managed to take blood after 60 s of their exposure to the blood exposition. The amount of blood consumed per fly was estimated as the difference in their weight, after (Supplementary Figure 2C) and before (Supplementary Figure 2A) the blood meal. As a control, we used fungus-free individuals. We collected all the data 2, 3, and 4 days after fungal infection. The feeding duration and the amount of blood consumed data were obtained from 30 fungus-exposed and fungus-free female flies; while the proportion of blood-fed data were from 5 groups of 10 individuals each.

### Influence of *M. anisopliae* ICIPE 30 on Gravid Female *S. calcitrans* Reproduction Traits

To elucidate whether *M. anisopliae* ICIPE 30 could affect the reproduction of *S. calcitrans*, we used (1) egg-laying decision, (2) fecundity and (3) fertility as proxies. To test the effect of *M. anisopliae* ICIPE 30 on *S. calcitrans* egg-laying decision, we conducted two oviposition choice bioassays (Supplementary Figure 3A). In the first bioassay, we exposed 10 gravid female *S. calcitrans* to two Petri dishes (Diameter: 5.5 cm) containing each, only 50 g of rabbit dung to see whether they will lay the same number of eggs on both Petri dishes. For the second bioassay, we presented rabbit dung supplemented with 0.1 g of *M. anisopliae* conidia (strain ICIPE 30) and rabbit dung only (control) to 10 gravid female *S. calcitrans* to see whether these females will select either substrate preferentially. In both bioassays, we used 10 replicates, and for each replicate, we counted the number of eggs laid on each substrate after 24 h and determined their ability to hatch 5 days after egg deposition (by counting the number of larvae found on each substrate).

To assess the effect of *M. anisopliae* ICIPE 30 on the fecundity (number of eggs laid) and fertility (number of eggs hatched) of *S. calcitrans*, we performed two no-choice oviposition bioassays (Supplementary Figures 3Bi,ii). To do so, following the previously described protocol, we exposed 30 females (4 days old) to *M. anisopliae* strain ICIPE 30 and transferred them individually inside cages (15 cm × 15 cm × 20 cm) containing 2 males to allow mating. We supplied these flies with blood daily and once females become gravid, we provided them with a Petri dish containing rabbit dung for oviposition. As a control, we used fungus-free gravid female *S. calcitrans*. We recorded the number of eggs laid on each substrate daily until the female succumbs from fungal infection. To assess the fertility of eggs laid by infected and uninfected gravid female *S. calcitrans*, we determined their hatchability by counting the number of larvae found on each substrate 5 days after the egg deposition.

### Trade-Offs Between *M. anisopliae* ICIPE 30 Infection Tolerance and Life-History Traits in *S. calcitrans* Larvae

It has been reported previously that *S. calcitrans* larvae are not susceptible to *M. anisopliae* infection (Moraes et al., 2008). To test this, we infected second larval instar of this fly with the same 11 strains of *M. anisopliae* as described above. For each strain, we sprayed 10 larvae (placed on a Petri dish) with 10 ml of suspension at the concentration of 2 × 10^8^ conidia ml^−1^ using a Burgerjon's spray tower (Burgerjon, 1956). After spraying, we transferred the contaminated larvae in transparent plastic cups of 200 ml prior containing 50 g of rabbit dung. As a control group, we used larvae treated with sterile distilled water containing 0.05% Triton X-100. We recorded the number of dead larvae daily until pupation. We carried out all the treatments in five times. We observed that most infected *S. calcitrans* larvae (90 %) managed to reach the pupal stage. Therefore, in a subsequent bioassay, we aimed to challenge these larvae with higher concentrations of *M. anisopliae*. As previously described, we contaminated 10 *S. calcitrans* larvae with four increasing conidia concentrations (3 × 10^8^, 4 × 10^8^, 5 × 10^8^, and 6 × 10^8^ conidia ml^−1^) of the strain ICIPE 30 and recorded the number of dead larvae daily until pupation.

To test whether the tolerance to *M. anisopliae* ICIPE 30 infection in *S. calcitrans* larvae could impact their fitness parameters, we contaminated 10 individuals of each *S. calcitrans* larval instar (L1, L2, and L3) with 10 ml of ICIPE 30 concentrated at 2 × 10^8^ conidia ml^−1^. It is indicated that life stages that undergo metamorphosis (occasioning the change in size and morphology) should be treated independently when studying their responses to biotic stresses (McCormick and Gagliano, 2009; Kingsolver et al., 2011; Ezeakacha and Yee, 2019). We followed the contaminated larvae daily until the adult stage by recording the following life-history fitness parameters: (1) pupation time, (2) larval weight, (3) pupation rate, (4) pupal weight, (5) emergence percentage, (6) emergence time, and (7) adult weight. Larval, pupal, and adult weight data were collected as described in Baleba et al. (2020). For the weight parameter, we weighed all the larvae individually, as well as pupae and adults that emerged from contaminated larvae. We recorded larval weight 2, 4, and 6 days after fungal contamination in the individuals from L1 and L2 instars. While in individuals from the L3 instar (close to the pupal stage), we recorded weight only 2 days after contamination. As a control, we used L1, L2, and L3 individuals sprayed with sterile distilled water containing 0.05% Triton X-100. We replicated this experiment five times.

### Data Analysis

We conducted all the statistical analysis in the R environment for statistical computing (version 3.6.3) (R Core Team., 2020) and grouped all the graphs in Adobe Illustrator CC 2017(version 21.0). Before conduct the analysis, we subjected mortality data to Abbot's correction (Abbot, 1925).

For the bioassay aiming to study the effect of the 11 strains of *M. anisopliae* on the *S. calcitrans* survival, we performed Kaplan–Meier survival analysis with the Mantel–Cox log-rank chi-squared test using the R package “survival” (Therneau, 2015) to see how the survival of *S. calcitrans* adults varied as a result to exposure to the different fungus strains. Owing to the normal distribution (Shapiro–Wilk test: *P* > 0.05) and the homoscedasticity (Bartlett's test: *P* > 0.05) of the median lethal time data, we ran the analysis of variance (ANOVA) followed by the Student–Neuman–Keuls (SNK) *post-hoc* multiple comparison tests to see how this parameter varied across the 11 strains. For the same reason, we performed the ANOVA followed by the SNK *post-hoc* tests to compare the proportion of alive *S. calcitrans* (at 7th day of our bioassay) across the 11 strains.

In the experiment testing the effect of sex and age on *M. anisopliae* infectivity, we used the Kaplan–Meier survival analysis with the Mantel–Cox log-rank chi-squared test to elucidate how these factors affected the infectivity of *M. anisopliae*. We employed the unpaired *t*-test to compare the median lethal time between the sexes of *S. calcitrans*. To determine whether this parameter could vary across the three ages of *S. calcitrans* (1, 7, and 14 days), we performed the ANOVA followed by the SNK *post-hoc* tests. Using the same analysis, we compared the number of alive *S. calcitrans* (at 7th day of our bioassay) across the sex and the ages.

For the experiment aiming to test whether, in *S. calcitrans, M. anisopliae* conidia could be transferred from one sex to another, we used the Kaplan–Meier survival analysis with the Mantel–Cox log-rank chi-squared test to see whether the survival of the fungus-donor, fungus-receiver, and fungus-free (control) *S. calcitrans* could significantly vary. We ran the unpaired *t*-test to compare the median lethal time between fungus-donor and fungus-receiver flies. We performed the ANOVA followed by the SNK *post-hoc* tests to compare the number of fungus-donor, fungus-receiver, and fungus-free *S. calcitrans* that were still alive at the end of our experiment (7th day).

In the feeding propensity test, we used the unpaired Wilcoxon test to compare the feeding time of infected and uninfected flies. Owing to the binary nature of the feeding proportion data (engorged vs. not engorged) we performed a generalized linear model (GLM) with binomial distribution followed by the analysis of deviance (with the chi-squared test) to see how the proportion of blood-fed flies varied between infected and uninfected flies. We executed the unpaired *t*-test to compare the amount of blood taken by infected and uninfected flies.

For the experiment testing the effect of *M. anisopliae* on *S. calcitrans* reproduction, we used the paired *t*-test to compare the number of eggs laid by gravid females *S. calcitrans* on the two Petri dishes containing only rabbit dung. We used the same statistical analysis to compare the number of eggs laid by these females on Petri dishes with and without *M. anisopliae* ICIPE 30 dried conidia. The Egg hatchability data were binary (hatched vs. unhatched); therefore, we used a GLM with binomial distribution and analysis of deviance (with chi-squared test) to see how this parameter varied between substrates with and without conidia. To compare the number of eggs laid by infected and uninfected gravid females *S. calcitrans*, we used an unpaired *t*-test. We compared the hatchability the eggs produced by these females, using a GLM with binomial distribution and analysis of deviance (with chi-squared test).

For the data from the bioassay testing the effect of *M. anisopliae* on the survival of *S. calcitrans* larvae, we performed the ANOVA test to compare the proportion of larvae that pupated across the different *M. anisopliae* strains and the ICIPE 30 concentrations. In the experiment testing the effect of *M. anisopliae* infection on the life-history parameters of three different larval instars of *S. calcitrans*, we subjected data from pupation time, larval weight, pupal weight, emergence time, and adult weight to the normality and homogeneity tests. In case the data of a particular parameter were normally distributed (Shapiro–Wilk test: *P* > 0.05) and their variances were homogeneous (Bartlett's test: *P* > 0.05), we used the unpaired *t*-test to see how this parameter varied between infected and uninfected larvae. When these two assumptions were not fulfilled, we used the unpaired Wilcoxon test. We analyzed the pupation and emergence percentage data using a GLM with binomial distribution and analysis of deviance (with chi-squared test).

Statistical significance was noted at *P* < 0.05 and its strength was represented with asterisks (^*^P < 0.05; ^**^P < 0.01; ^***^P < 0.001, and ^****^P < 0.001).

## Results

### Effect of *M. anisopliae* Infection on the Survival of *S. calcitrans* Adults

**Figure 1:**
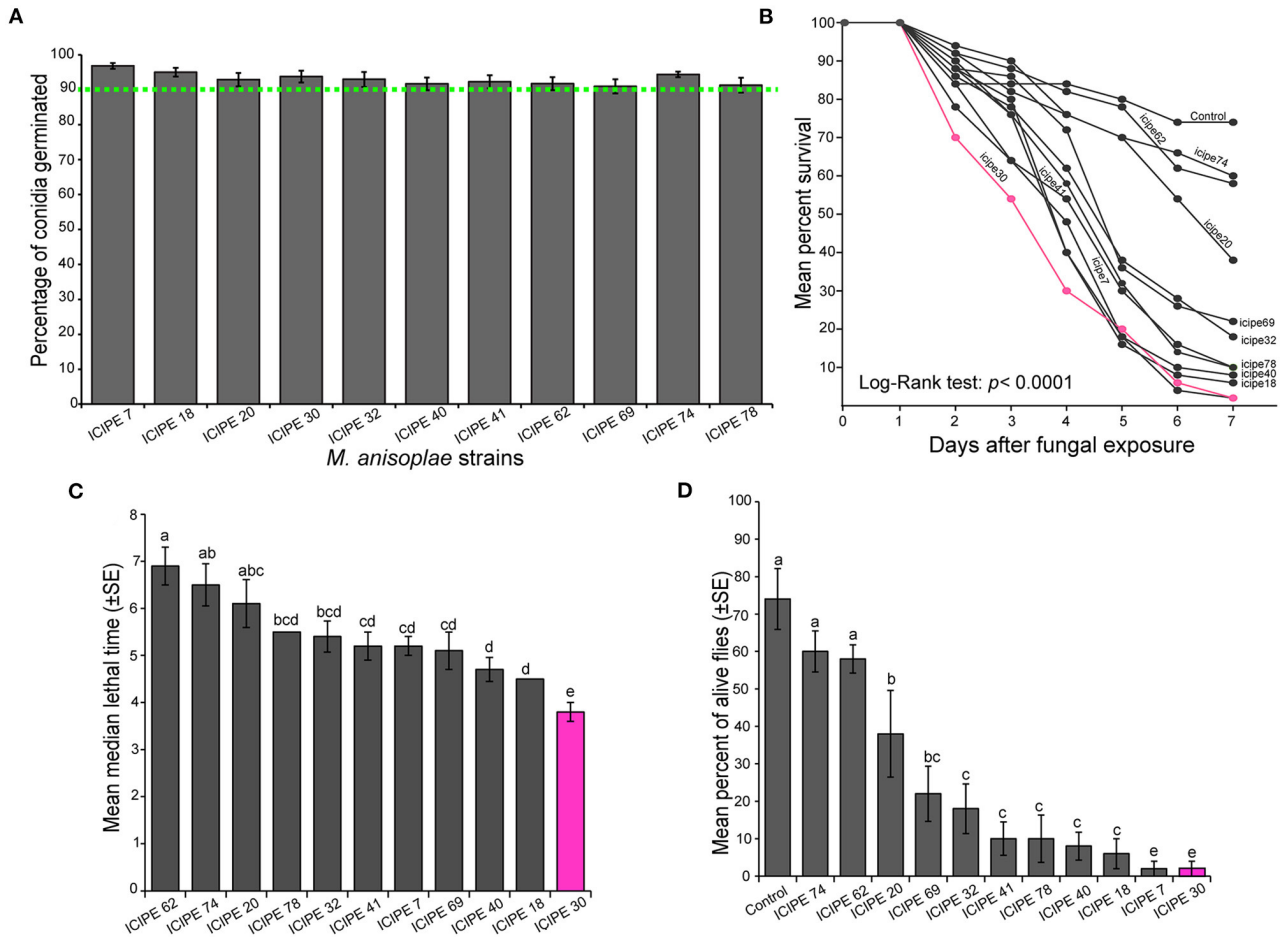
Virulence of different *Metarhizium anisopliae* strains toward *Stomoxys calcitrans*. **(A)** Bar chart showing the mean percentage germination of the 11 *M. anisopliae* strains used in all our experiment. **(B)** Kaplan–Meier curve showing survivorship over time in the adult of *S. calcitrans* across the different *M. anisopliae* strains (Mantel–Cox log-rank χ^2^ test, *P* < 0.05, *n* = 5). **(C)** Bar chart illustrating the mean median lethal time (LT_50_) in *S. calcitrans* adults infected by the different *M. anisopliae* strains. **(D)** Bar chart depicting the mean percentage of *S. calcitrans* adults alive 7 days after fungal infection, across the different *M. anisopliae* strains (ANOVA followed by SNK *post-hoc* test; *p* < 0.05, *n* = 5). Error bars indicate the standard error of the mean. Bars with different lowercase letters are significantly different from each other.

All the 11 strains of *M. anisopliae* used in our study possessed a germination percentage above 90% (Figure 1A). As time progressed, the proportion of *S. calcitrans* surviving from *M. anisopliae* infection reduced with a significant difference across the strains (Figure 1B; log-rank test, χ^2^ = 50.3, df = 11, *P* < 0.0001). The median lethal time [Figure 1C; One-way ANOVA: *F*_(10−44)_ = 7.79, *P* < 0.0001] and the proportion of alive *S. calcitrans* after the 7 days post-infection [Figure 1D; One-way ANOVA: *F*_(11−47)_ = 17.5, *P* < 0.0001] significantly differed across the 11 strains of *M. anisopliae*. Of all the *S. calcitrans* individuals infected with 11 strains of *M. anisopliae*, only those infected with the strain ICIPE 30 had simultaneously, lower median lethal time (Figure 1C) and lower proportion of alive individuals (20%) at the end of our experiment (Figure 1D). Although at the end of our experiment, the proportion of alive *S. calcitrans* infected with the strain ICIPE 7 was similar to that of *S. calcitrans* infected with the strain ICIPE 30 (Figure 1D), the strain ICIPE 7 took the longest time (>5 days) to kill half individuals of *S. calcitrans* as compared to the strain ICIPE 30 (<4 days) (Figure 1C). Therefore, we considered the strain ICIPE 30 as the most potent and virulent for *S. calcitrans*.

### Effect of *S. calcitrans* Sex and Age on *M. anisopliae* ICIPE 30 Infectivity

**Figure 2:**
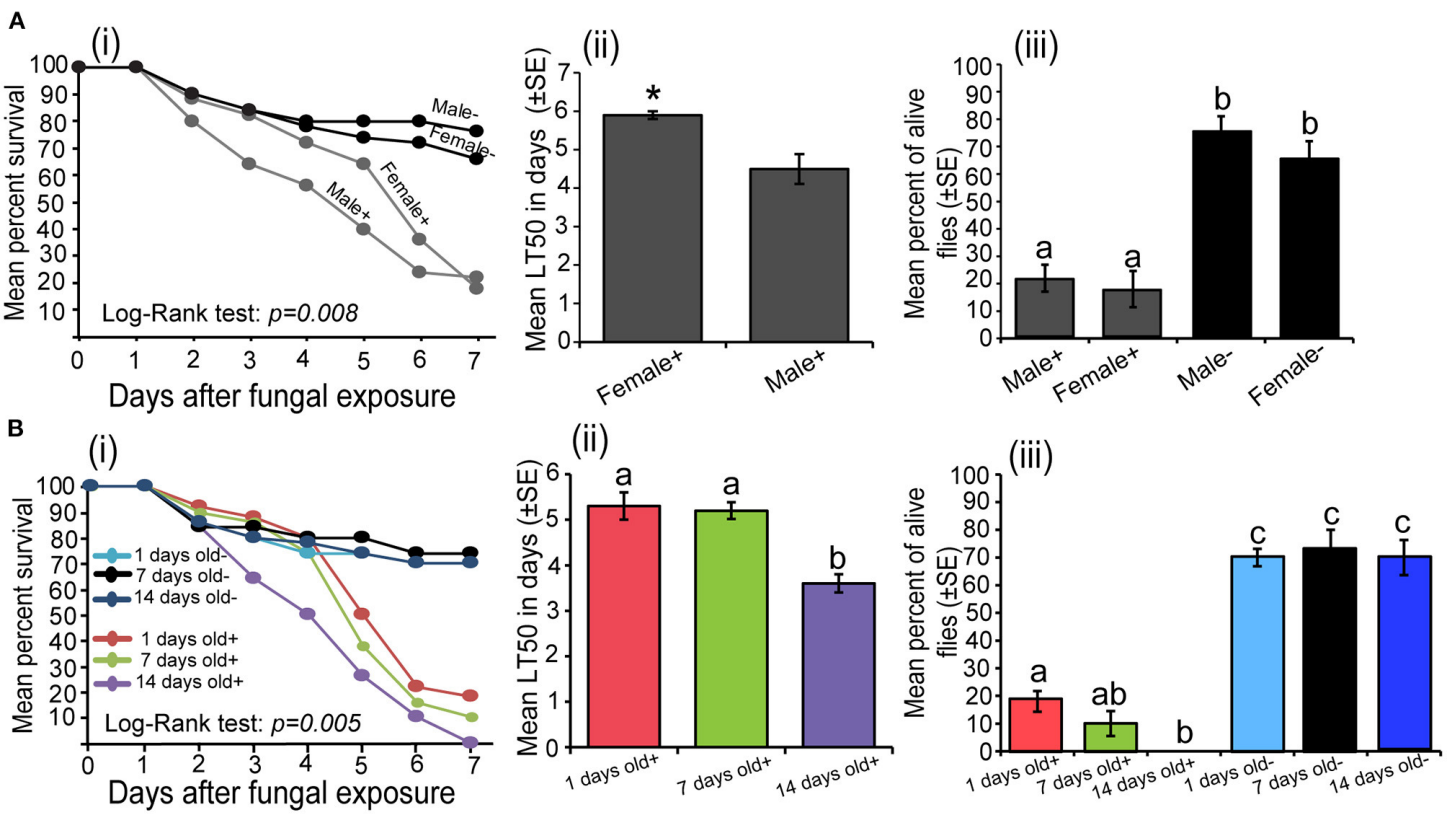
**(A)** Influence of the *Stomoxys calcitrans* sex on *Metarhizium anisopliae* ICIPE 30 infectivity: (i) Kaplan–Meier curve showing survivorship over time of infected (males and females) and uninfected (males and females) *S. calcitrans* (Mantel–Cox log-rank χ^2^ test, *P* < 0.05, *n* = 5), (ii) Bar chart illustrating the mean median lethal time (LT_50_) between infected males and females of *S. calcitrans* (unpaired *t*-test, *p < *0.05, n* = 5), (iii) Bar chart depicting the mean percentages of individual alive across the sex of infected and uninfected *S. calcitrans* the 7th day of our bioassay (ANOVA followed by SNK *post-hoc* test; *p* < 0.05, *n* = 5). **(B)** Influence of the *S. calcitrans* age on *M. anisopliae* ICIPE 30 infectivity: (i) Kaplan–Meier curve showing survivorship over time of infected (1, 7, and 14 days old) and uninfected (1, 7, and 14 days old) *S. calcitrans* (Mantel–Cox log-rank χ^2^ test, *P* < 0.05, *n* = 5), (ii) Bar chart illustrating the mean median lethal time (LT_50_) across the age of infected *S. calcitrans* (ANOVA followed by SNK *post-hoc* test; *p* < 0.05, *n* = 5), (iii) Bar chart showing the mean percentages of individual alive across the age of infected and uninfected *S. calcitrans* at the 7th day of our bioassay (ANOVA followed by SNK *post-hoc* test; *p* < 0.05, *n* = 5). The signs + and – denote infected and uninfected individuals, respectively. Error bars indicate the standard error of the mean. Bars with different lowercase letters are significantly different from each other.

In both sex of *S. calcitrans*, the survival over time of uninfected (males and females) and infected (males and females) individuals significantly varied (Figure 2Ai; log-rank test, χ^2^ = 11.8, df = 3, *P* = 0.008). But the pairwise comparison using the log-rank test revealed that the survival of infected males and infected females over time did not significantly differ (*P* = 0.26). This was also true for uninfected males and uninfected females (*P* = 0.39). The mean median lethal time of infected female *S. calcitrans* was significantly higher than that of infected males (Figure 2Aii; *U* = 22, *P* = 0.04). At the end of our experiment, infected flies of both sexes had a significantly lower proportion of alive individuals as compared to that uninfected flies [Figure 2Aiii; One-way ANOVA: *F*_(3−16)_ = 27.27, *P* < 0.0001]. But this proportion was similar between infected males and females; and uninfected males and females.

The survival of *S. calcitrans* over time significantly changed across the different age of flies (log-rank test, χ^2^ = 16.9, df = 5, *P* = 0.005); with 14-days old infected possessing the lower survival rate (Figure 2Bi). As compared to 1 and 7 days old, 14-days old infected flies had a smaller median lethal time [Figure 2Bii; One-way ANOVA: *F*_(2−12)_ = 16.65, *P* < 0.001]. Regardless of the age, the proportion of alive flies obtained 7 days after contamination was significantly lower in infected flies and as compared to uninfected flies [Figure 2Biii; One-way ANOVA: *F*_(5−24)_ = 59.39, *P* < 0.0001]. In infected flies, this proportion was significantly lower in 14 days old flies followed by 7 and 1-day old flies (Figure 2Biii).

### Horizontal Transmission of *M. anisopliae* ICIPE 30 Conidia by *S. calcitrans*

**Figure 3:**
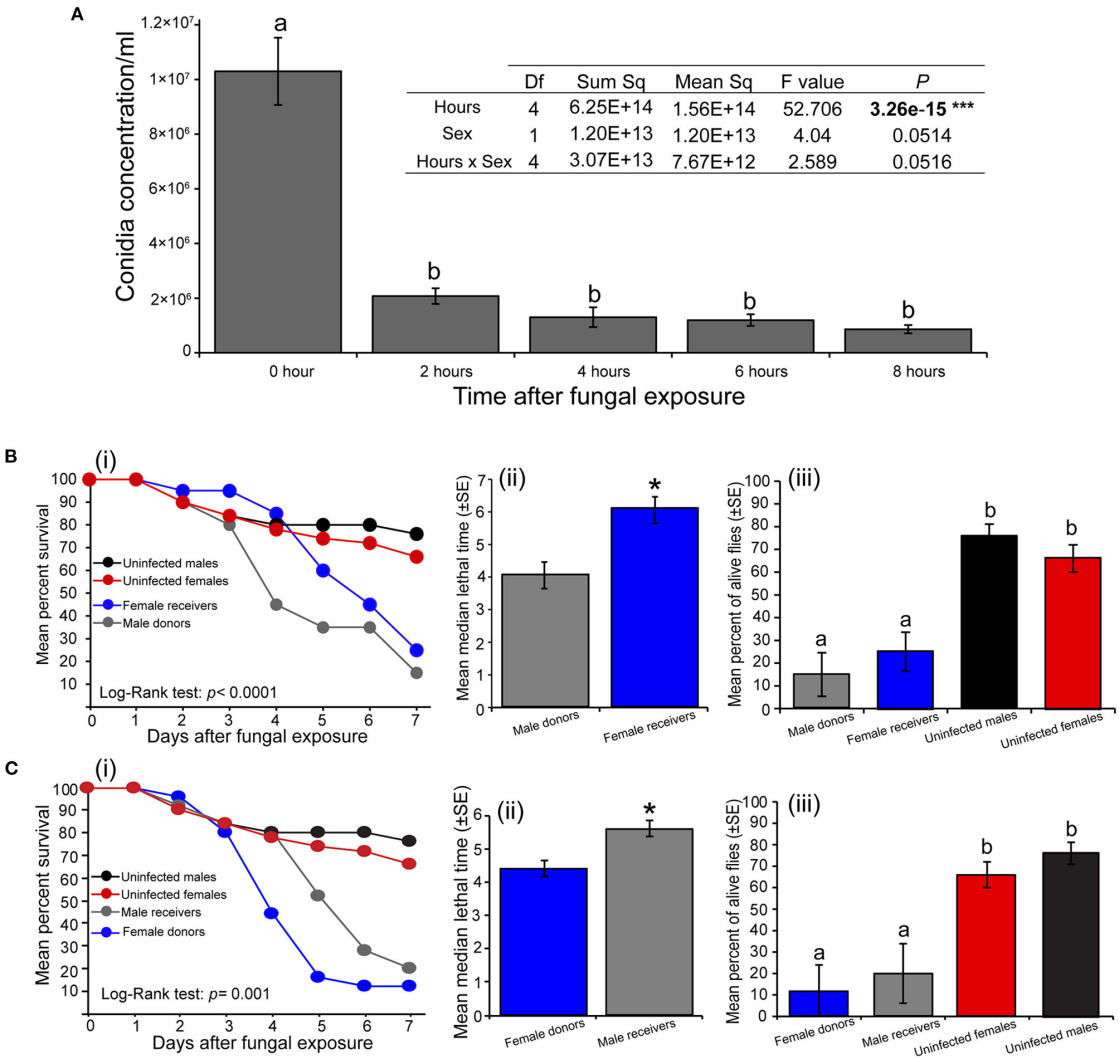
Horizontal transmission of *Metarhizium anisopliae* conidia in *Stomoxys calcitrans*. **(A)** Bar chart showing the variation across the time of conidia load in *S. calcitrans* (one-way ANOVA followed by SNK *post-hoc* test; *P* < 0.05, ***P < 0.001, *n* = 10). **(B)** Transmission of *M. anisopliae* ICIPE 30 conidia from *S. calcitrans* males (donors) to females (receivers): (i) Kaplan-Meier curve showing survivorship over time of *S. calcitrans* male donors and female receivers (Mantel-Cox log-rank χ^2^ test, *P* < 0.05, *n* = 5); (ii) Bar chart illustrating the mean median lethal time (LT_50_) of male donors and female receivers (unpaired *t*-test, *P < 0.05, *n* = 5); (iii) Bar chart illustrating the mean percentage of *S. calcitrans* male donors and female receivers alive the 7th day of our bioassay (one-way ANOVA followed by SNK *post-hoc* test; *P* < 0.05, *n* = 5). **(C)** Transmission of *M. anisopliae* ICIPE 30 conidia from *S. calcitrans* females (donors) to males (receivers): (i) Kaplan–Meier curve showing survivorship over time of *S. calcitrans* female donors and male receivers (Mantel–Cox log-rank χ^2^ test, *P* < 0.05, *n* = 5); (ii) Bar chart illustrating the mean median lethal time (LT_50_) of female donors and male receivers (unpaired *t*-test, *P < 0.05, *n* = 5); (iii) Bar chart depicting the mean percentage of *S. calcitrans* female donors and male receivers alive the 7th day of our bioassay (ANOVA followed by SNK *post-hoc* test; *P* < 0.05, *n* = 5). Error bars indicate the standard error of the mean. Letters above error bars indicate a significant difference across the treatment.

The amount of *M. anisopliae* ICIPE 30 conidia carried by *S. calcitrans* significantly varied across time (*P* < 0.0001) with no variations between sex (*P* = 0.051). This amount was higher directly after the contamination process; but 2 h later, it drastically dropped with no significant change even 8 h after the fly's contamination (Figure 3A). We found that 4 h after exposure to *M. anisopliae* ICIPE 30 conidia, fungus-exposed flies (donors) were still able to contaminate fungus-free flies (receivers). When we contaminated *S. calcitrans* males (donors) and associated them with fungus-free females (receivers), the survival of these flies over time significantly reduced as compared to those of males and females maintained uncontaminated throughout the bioassay (Figure 3Bi; log-rank test, χ^2^ = 26.2, df = 3, *P* < 0.0001). The median lethal time of male donors was significantly lower as compared to that of female receivers (Figure 3Bii; *t* = 3.6, df = 6, *P* = 0.013). Both male donors and female receivers had reduced proportion of alive individuals at the 7th-day post-contamination as compared to males and females maintained uncontaminated [Figure 3Biii; One-way ANOVA: *F*_(3−14)_ = 16.29, *P* < 0.0001]. We obtained the same result pattern when we contaminated females (donors) and associated them with fungus-free males (receivers). The survival of female donors and male receivers significantly reduced over time as compared to the survival of uncontaminated males and females (Figure 3Ci; log-rank test, χ^2^ = 16.2, df = 3, *P* < 0.001). Female donors had a lower mean median lethal time as compared to that of male receivers (Figure 3Cii, *U* = 2, *P* = 0.026). The proportion of female donors and male receivers alive at the end of our experiment was significantly lower than those of uncontaminated males and females [Figure 3Ciii; One-way ANOVA: *F*_(3−17)_ = 10.2, *P* < 0.001].

### Effect of *M. anisopliae* ICIPE 30 Infection on the Feeding Propensity of *S. calcitrans*

**Figure 4:**
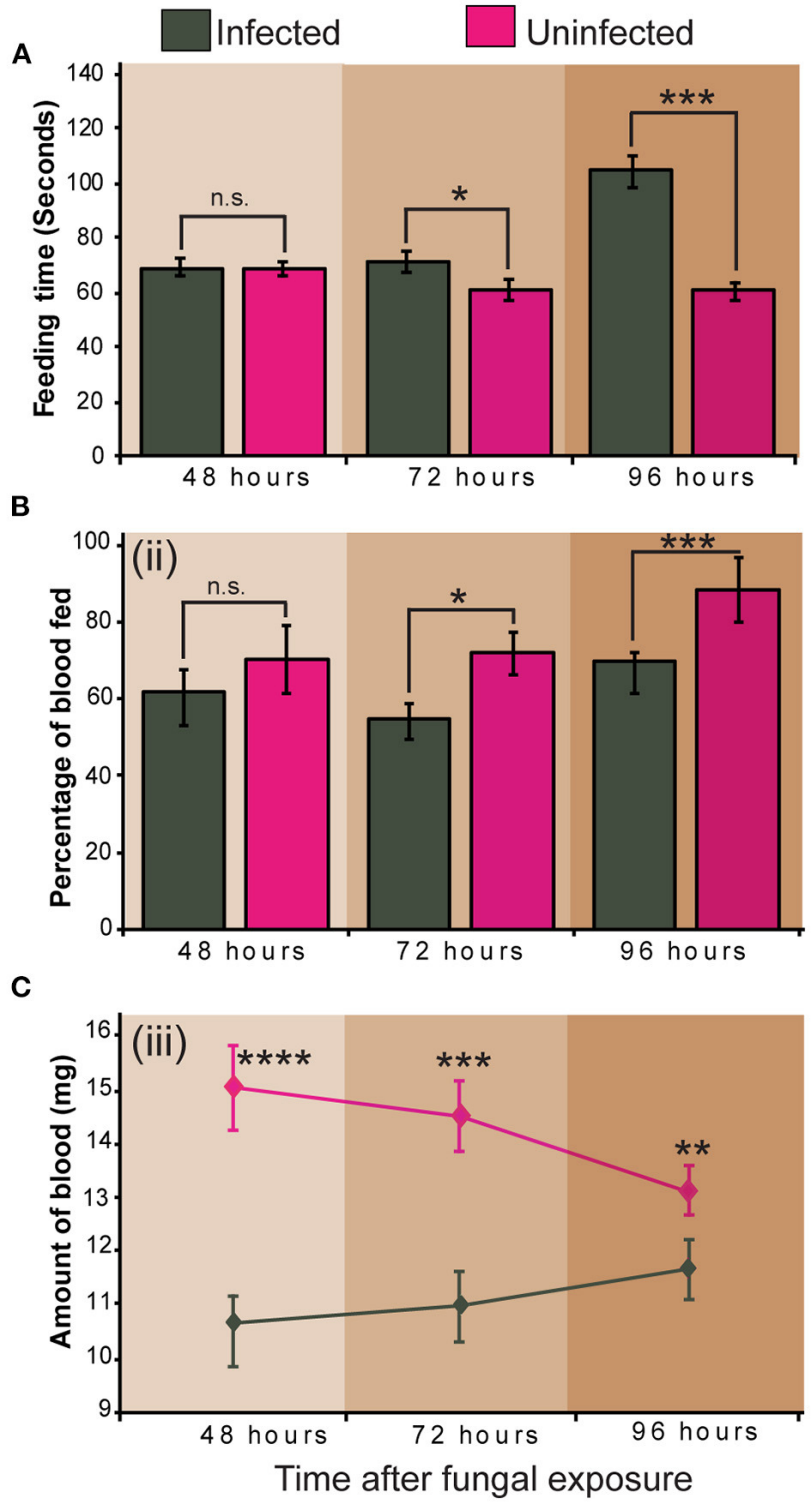
Impact of *Metarhizium anisopliae* infection on *Stomoxys calcitrans* feeding propensity. **(A)** Bar charts showing the mean feeding duration across time in infected and uninfected *S. calcitrans*; **(B)** Bar charts illustrating the mean percentage of infected and uninfected *S. calcitrans* individuals that managed to get engorged in 60 s across time; **(C)** Line graph showing the change of the amount of blood taken by infected and uninfected *S. calcitrans* across time. Error bars indicate the standard error of the mean. Asterisks indicate that mean between infected and uninfected individuals differed significantly (unpaired *t*-test: *P < 0.05; **P < 0.01, ***P < 0.001, ****P < 0.0001, *n* = 30). “n.s” indicates non-significant difference (*P* > 0.05).

Infection of *S. calcitrans* by *M. anisopliae* ICIPE 30 significantly altered its feeding propensity. Fourty-eight (48) hours after fungal exposure, the time taken by *S. calcitrans* to get engorged did not vary between infected and uninfected flies (*t* = 0.06, df = 67.7, *P* = 0.95). But, 72 (*t* = 2.18, df = 65.5, *P* = 0.032) and 96 (*U* = 466, *P* < 0.001) h after the conidia exposure occurred, infected flies took significantly more time to get engorged as compared to uninfected flies (Figure 4A). Sixty (60) seconds after blood exposure, 48-h infected flies and uninfected flies had the same proportion flies that managed to take blood (Figure 4Bii; GLM, χ^2^ = 19.92, df = 1, *P* = 0.50). Nonetheless, this proportion significantly reduced in 72-h (GLM, χ^2^ = 9.5, df = 1, *P* = 0.042) and 96-h (GLM, χ^2^ = 25.53, df = 1, *P* = 0.004) infected flies (Figure 4B). The amount of blood taken by infected flies at 48 (*t* = 4.60, df = 61.5, *P* < 0.0001), 72 (*t* = 3.71, df = 66, *P* < 0.001), and 96 (*t* = 2.9, df = 39.4, *P* < 0.01) h after fungal exposure was significantly lower as compared to that of uninfected flies (Figure 4C).

### Influence of *M. anisopliae* ICIPE 30 on *S. calcitrans* Female's Reproduction Traits

**Figure 5:**
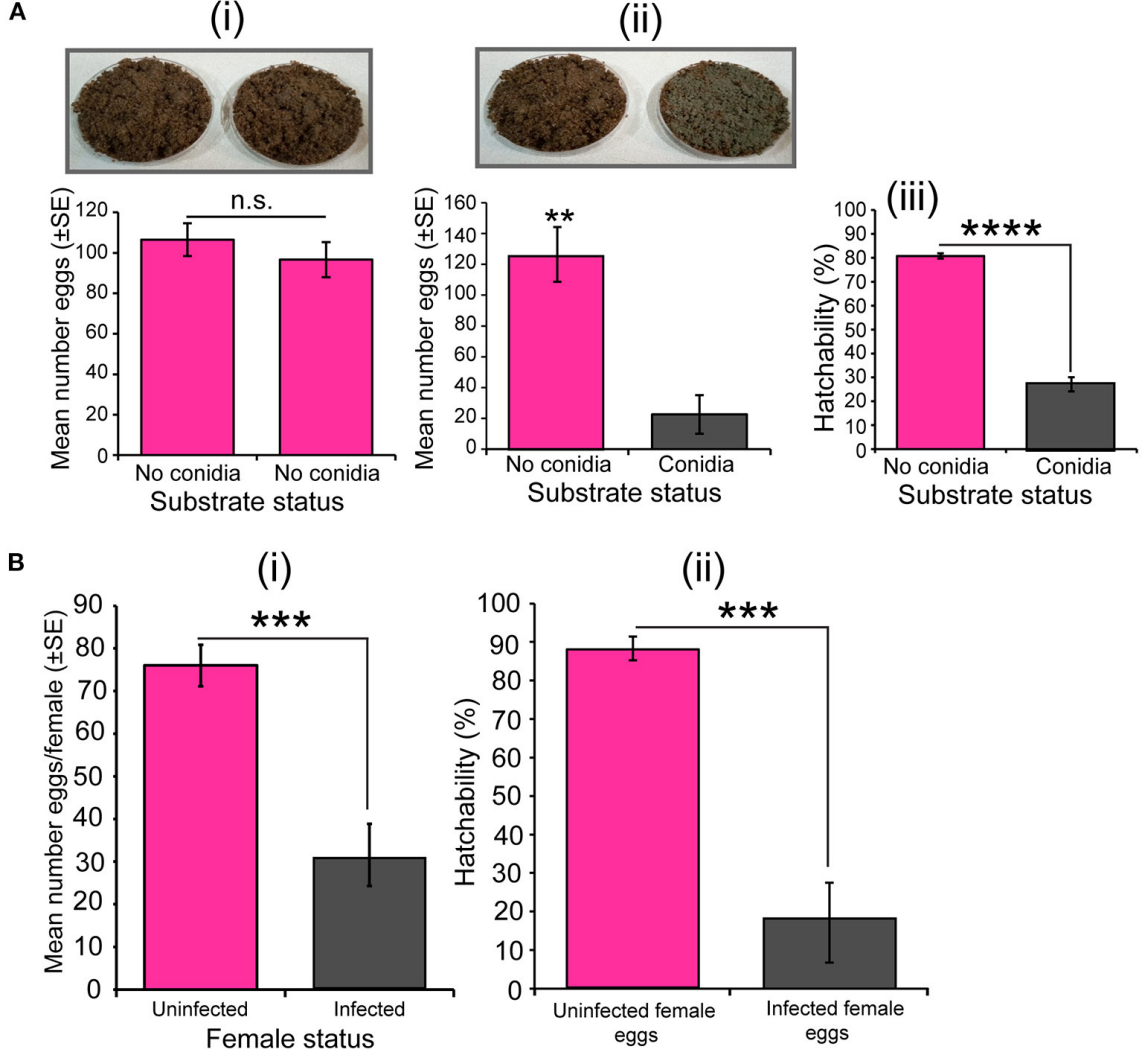
Effect of *Metarhizium anisopliae* on the oviposition decision, fecundity, and egg hatchability of *Stomoxys calcitrans*. **(A)** Egg-laying decision bioassay with uninfected gravid female *S. calcitrans*: (i) Bar chart showing the mean number of eggs laid by *S. calcitrans* on substrates without dried conidia of *M. anisopliae* ICIPE 30 (paired *t*-test, *n* = 10); (ii) Bar chart showing the mean number of eggs laid by *S. calcitrans* on substrates with and without dried conidia of *M. anisopliae* ICIPE 30 (paired *t*-test: *p* > 0.05, *n* = 10); (iii) Bar chart illustrating the proportion of eggs hatched from eggs laid of substrates with and without dried conidia of *M. anisopliae* ICIPE 30 (GLM with binomial distribution followed by the analysis of deviance test, *n* = 10). **(B)** Egg-laying assay with single uninfected and infected gravid female *S. calcitrans*. (i) Bar chart illustrating the mean number of eggs laid by uninfected and infected gravid females of *S. calcitrans* (unpaired *t*-test, *n* = 30); (ii) Bar chart showing the mean proportion of eggs that hatched from eggs laid by uninfected and infected of *S. calcitrans* (GLM with binomial distribution followed by the analysis of deviance test). Errors bar on each bar chart indicates the standard error of the mean. **P < 0.01, ***P < 0.001, and ****P < 0.0001.

In our dual-test oviposition bioassay, when we presented two fungus-free substrates to *S. calcitrans* gravid females, they laid the same number of eggs on both substrates (Figure 5Ai, *t* = 0.83, df = 17.9, *P* = 0.41). However, when we added dried conidia of *M. anisopliae* on one of the substrates, these females laid significantly few numbers of eggs on the fungal-embedded substrate (Figure 5Aii; *U* = 0, *P* < 0.01). Consequently, the proportion of eggs deposited on the substrate with dried conidia that hatched was significantly less than that of eggs laid on the substrate without dried conidia (Figure 5Aiii, GLM; χ^2^ = 23.07, df = 1, *P* < 0.0001). In the no-choice bioassay, *S. calcitrans* gravid females infected with *M. anisopliae* ICIPE 30 laid a significantly fewer number of eggs than uninfected *S. calcitrans* gravid females (Figure 5Bi; *t* = 5.08, df = 11.84, *P* < 0.001). Also, the hatchability of eggs laid by infected females was significantly lower than that of eggs deposited by uninfected females (Figure 5Bii, GLM; χ^2^ = 26.63, df = 1, *P* < 0.0001).

### Fitness Cost Associated With the Tolerance of *M. anisopliae* Infection in *S. calcitrans* Larvae

**Figure 6:**
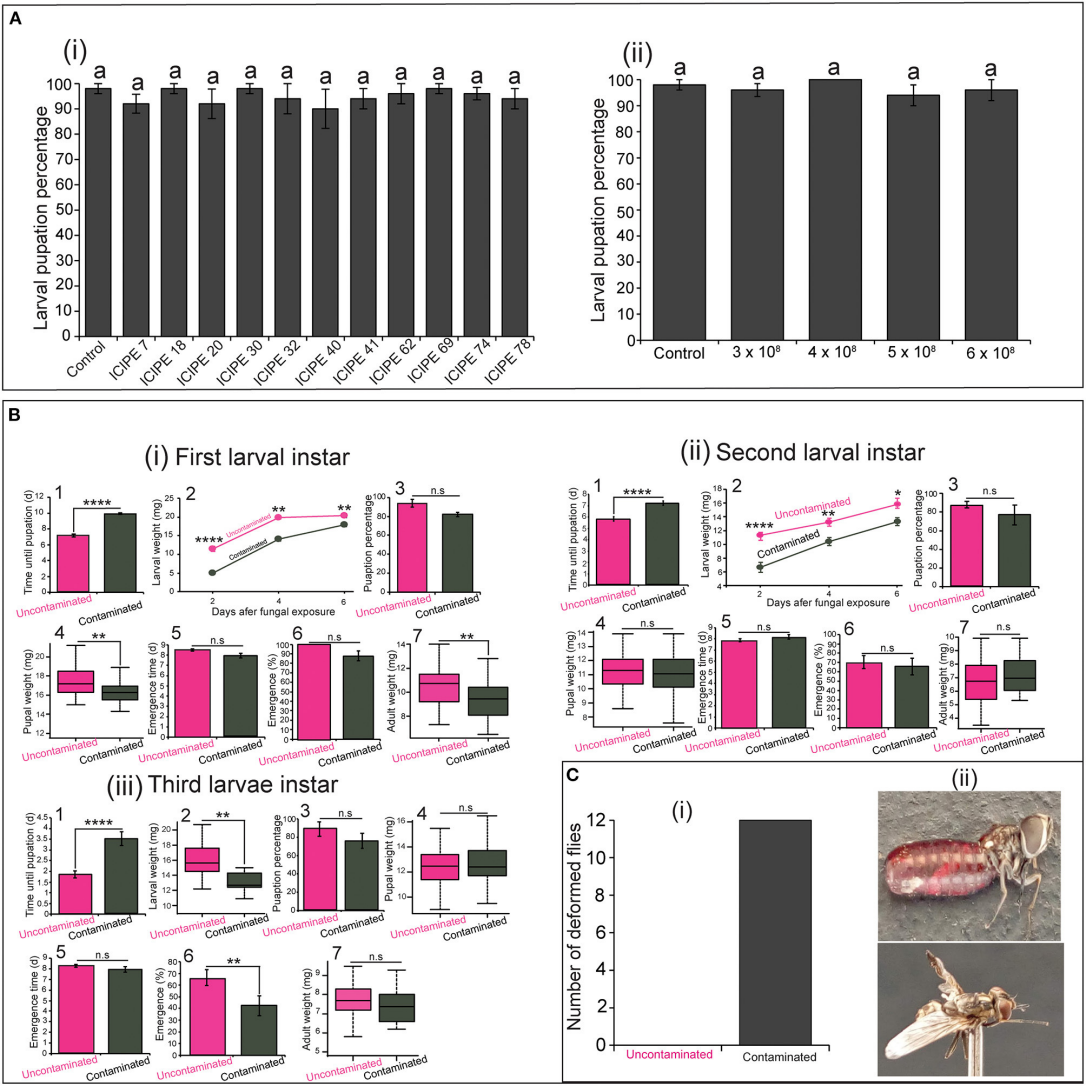
Influence of *Metarhizium anisopliae* infection on the fitness parameters of the three larval instars of *Stomoxys calcitrans*. **(A)** Bar chart showing the mean pupation percentage of *S. calcitrans* larvae contaminated by the 11 strains of *M. anisopliae* (i) and the increasing concentrations of the *M. anisopliae* strain ICIPE 30 (ii) (ANOVA test; *n* = 5). **(B)** Fitness parameters of uncontaminated and contaminated first (i), second (ii), and third (iii) larval instar of *S. calcitrans*: (1) Bar charts depicting the mean pupation time in each uncontaminated and contaminated larval instar of *S. calcitrans* (unpaired Mann–Whitney test); (2) Line graphs showing the variation of weight in each uncontaminated and contaminated larval instar of *S. calcitrans* after 2, 4, and 6 days of infection (unpaired *t*-test test or unpaired Mann–Whitney test); (3) Bar charts illustrating the mean pupation percentage of each uncontaminated and contaminated larval instar of *S. calcitrans* (GLM with binomial distribution followed by the analysis of deviance test, *n* = 5); (4) Box plots showing the pupal weight obtained from each uncontaminated and contaminated larval instar of *S. calcitrans* (unpaired *t*-test); (5) Bart charts illustrating the mean emergence time of *S. calcitrans* adults developed from each uncontaminated and contaminated larval instars (unpaired Mann–Whitney test); (6) Bar charts depicting the emergence percentage of *S. calcitrans* adults developed from each uncontaminated and contaminated larval instars (GLM with binomial distribution followed by the analysis of deviance test, *n* = 5); (7) Box plots showing the weight of *S. calcitrans* adults developed from each uncontaminated and contaminated larval instars (unpaired *t*-test). **(C)** Histogram (i) showing the total number of deformed *S. calcitrans* adults (ii; Original Photo: Steve B. S. Baleba) emerged from contaminated larvae. On each bar chart and line graph, error bars indicate the standard error of the mean. Each box plots shows the median (bold horizontal lines) and whiskers the interquartile range. n.s (non-significant difference): *P* > 0.05; significant difference: *P < 0.05, **P < 0.01, and ****P < 0.0001.

As demonstrated in other studies, our study showed that *S. calcitrans* larvae are not susceptible to infection with *M. anisopliae* strains used. All the *M. anisopliae* strains-contaminated larvae and uncontaminated larvae (control) had similar pupation percentage [*F*_(11−48)_ = 0.42, *P* = 0.94]. For each treatment, about 90% of larvae reached the pupal stage (Figure 6Ai). Even at high conidia concentrations, the pupation percentage of contaminated larvae did not reduce [Figure 6Aii; *F*_(4−20)_ = 0.62, *P* = 0.65]. Even though the high proportion of contaminated larvae did not succumb from *M. anisopliae* exposure (since some managed to pupate), we demonstrated a negative impact of this contamination on some of their fitness parameters.

We observed that the pupation time was significantly longer in contaminated first (Figure 6Bi1: *U* = 70, *P* < 0.0001), second (Figure 6Bii1: *U* = 255.5, *P* < 0.0001), and third (Figure 6Biii1, *U* = 346, *P* < 0.0001) larval instars of *S. calcitrans* as compared to that of their corresponding control (uncontaminated larvae). Also, the weight of contaminated first (Figure 6Bi2: Day 2: *t* = 8.45, df = 14.4, *P* < 0.0001; Day 4: *U* = 317, *P* = 0.002; Day 6: *t* = 3.2, df = 17.98, *P* = 0.005), second (Figure 6Bii2: Day 2: *t* = 5.5, df = 14.6, *P* < 0.0001, Day 4: *t* = 3.5, df = 18, *P* = 0.002; Day 6: *t* = 2.4, df = 15.9, *P* = 0.026), and third (Figure 6Biii2; *t* = 2.97, df = 15.4, *P* = 0.009) larval instar of *S. calcitrans* was smaller than those of uncontaminated instars. The pupation percentage did not significantly change between contaminated and uncontaminated individuals from the first (Figure 6Bi3; GLM; χ^2^ = 3.55, df = 1, *P* = 0.06), second (Figure 6Bii3; GLM; χ^2^ = 3.25, df = 1, *P* = 0.071), and third (Figure 6Biii3; GLM; χ^2^ = 2.48, df = 1, *P* = 0.11) larval instars. The pupal weight was significantly reduced in contaminated individuals developed from the first larval instars (Figure 6Bi4: *t* = 3.5, df = 80, *P* < 0.001). However, pupae formed from the contaminated second (Figure 6Bii4: *t* = −0.73, df = 37.66, *P* = 0.46), and third (Figure 6Biii4: *t* = −0.89, df = 65.51, *P* = 0.37) larval instars had similar weight with those developed from uncontaminated second and third larval instars respectively. Pupae developed from contaminated and uncontaminated first (Figure 6Bi5: *U* = 1002.5, *P* = 0.12), second (Figure 6Bii5: *U* = 229.5, *P* = 0.16), and third (Figure 6Biii5: *U* = 262.5, *P* = 0.32) larval instars formed at the same time. The proportion of adults obtained from contaminated and uncontaminated first (Figure 6Bi6: GLM; χ^2^ = 1.46, df = 1, *P* = 0.22) and second (Figure 6Bii6: GLM; χ^2^ = 0.002, df = 1, *P* = 0.97) larval instars was not significantly different; but in the third larval instar, this proportion was significantly higher in uncontaminated larvae (Figure 6Biii6: GLM; χ^2^ = 7.04, df = 1, *P* = 0.008). Adult obtained from contaminated first larval instars had a significant reduced weight as compared to those emerged from uncontaminated first larval instars (Figure 6Bi7: *t* = −3.27, df = 68.8, *P* = 0.002). While those obtained from contaminated and uncontaminated second (Figure 6Bii7: *t* = −1.06, df = 44.2, *P* = 0.29) and third (Figure 6Biii7: *t* = 1.27, df = 29.45, *P* = 0.21) larval instars had the same weight. Independently to the larval instars, we numbered 12 deformed adults from contaminated larvae and no deformed adults from uncontaminated larvae (Figure 6C).

## Discussion

Our results demonstrate that *M. anisopliae* infection significantly reduces the survival, feeding propensity, fecundity, and fertility of *S. calcitrans*. Also, these findings show that there is a fitness cost associated with the tolerance of *S. calcitrans* larvae to *M. anisopliae* infection.

All the 11 strains of *M. anisopliae* studied killed *S. calcitrans* adults; although the speed and the rate at which this occurred varied significantly among the strains. Several studies have demonstrated the implication of morphological and physiological characteristics of fungal strains on this virulence variation. These characteristics include hyphal growth rate, conidial viability, conidia production, conidia size, enzyme secretion among other factors (Liu et al., 2003; Quesada-Moraga and Vey, 2003; Talaei -Hassanloui et al., 2006). In our study, *M. anisopliae* strain ICIPE 30 rapidly killed (<4 days) half of *S. calcitrans* individuals and appeared to be the most virulent strain. The virulence of this strain has previously been demonstrated in other haematophagous dipterans including *Glossina morsitans morsitans* (Maniania and Odulaja, 1998), *An. gambiae* (Mnyone et al., 2009, 2011) and *Ae. aegypti* (Jemberie et al., 2018). At this stage, there is a need to study the genetic, molecular and physiological mechanisms mediating the virulence of ICIPE 30. Niassy et al. (2013) started by characterizing the chitinase genes (chi2 and chi4) of this strain responsible for the secretion of enzymes that digest the insect cuticle. The overexpression of such genes in *Metarhizium* fungus using bioengineering methods could increase their virulence and this need to be investigated further. For instance, the genetically engineered *M. anisopliae* in which the *cat1* gene has been overexpressed tolerate more exogenous hydrogen peroxide; resulting in the acceleration of its germination and the increase of its virulence (Morales Hernandez et al., 2010).

We demonstrated that the *M. anisopliae* infectivity could be influenced by the sex and the age of *S. calcitrans* individuals. As compared to female *S. calcitrans, M. anisopliae* ICIPE 30 killed faster 50 % of male *S. calcitrans* (Figure 2Aii). This may indicate that there are differences in the innate immunity of male *S. calcitrans* compared to females. We speculate that female *S. calcitrans* produce more vigorous cellular and humoral immune reactions against *M. anisopliae* blastospore. Using *Drosophila melanogaster* (Diptera: Drosophilidae) and *Beauveria bassiana* (Hypocreales: Cordycipitaceae), Shahrestani et al. (2018) demonstrated the existence of sexual dimorphism in the immune response of insects to fungal infection. Our results contrast with those of Maniania and Odulaja (1998) who showed that females of *G. m. morsitans* and *G. m. centralis* succumb first to ICIPE 30 infection. We attribute this difference to the dissimilarity of the insect species used in our respective studies. Nonetheless, our finding is supported by other studies involving different strains of *M. anisopliae* and fungal species. Kaaya (1989) showed that males of *G. m. morsitans* infected with *M. anisopliae* strains 35-79, 82-82, and 100-82 were more susceptible to the infection than females. In *Musca domestica* (Diptera: Muscidae), males infected with *Entomophthora muscae* (Entomophthorales: Entomophthoraceae) died significantly earlier than females (Mullens, 1985). In addition to this effect of sex, we also found a significant effect of the *S. calcitrans* age on *M. anisopliae* ICIPE 30 infectivity. Fourteen-days old female *S. calcitrans* infected with ICIPE 30 died relatively sooner than females of 1 and 7-days old. This increase of mortality with the age in infected *S. calcitrans* has already been observed in other blood-feeding insects including adult tsetse flies (Maniania and Odulaja, 1998) and *An. gambiae* (Mnyone et al., 2011). The reduction of the immune response to *M. anisopliae* infection could explain why older female *S. calcitrans* died faster. In general, the immune system of animals weakens as they become older. For instance, the melanization which is an immediate immune response to pathogens in arthropods is reduced in old *Ae. aegypti* individuals (Christensen et al., 1986). Enzymes such as phenoloxidases which play a key role in insect immune system decline with the insect age (González-Santoyo and Córdoba-Aguilar, 2012). In *Ae. aegypti*, the high mortality observed in old individuals after their infection with *Escherichia coli* is associated to decrease in the number of hemocytes in their hemolymph (Hillyer et al., 2005). Our results would be explained better by further studies investigating the change that undergoes the immune system of *S. calcitrans* with the age.

We showed that the number of conidia attached to *S. calcitrans* cuticle reduced drastically after the exposure (~75% within 2 h) (Figure 3A). This result could be associated with the fact that, after the fungal exposure, *S. calcitrans* exhibit active grooming behavior, with flies trying to clean as much as possible all their body parts. The conidia found on *S. calcitrans* 8 h after exposure could be from the areas hard to reach (e.g., back of thorax and abdomen) during the active grooming. This reduction of the conidia load over time has also been found in *Ceratitis cosyra* (Diptera: Tephritidae) (Dimbi et al., 2013). We demonstrated that in *S. calcitrans*, 4 h after *M. anisopliae* contamination, conidia of this fungus can still be transmitted horizontally from one sex to another. The survival of females that received ICIPE 30 conidia from contaminated males was significantly lower than that of uncontaminated females (Figure 3B). We obtained a similar result with males that received conidia from contaminated females (Figure 3C). Horizontal transmission of fungal conidia is also found in other blood-feeding insect species including *G. morsitans* (Maniania et al., 2013), *Triatoma infestans* (Hemiptera: Rediviidae) (Forlani et al., 2011), *An. gambiae* (Scholte et al., 2004) and *Ae. aegyptis* (Diptera: Culicidae) (García-Munguía et al., 2011). We hypothesize that the horizontal transmission of *M. anisopliae* conidia in *S. calcitrans* occurs during courtship events including wing extension and vibration, abdomen drumming and curving, or proboscis extension. Also, the fact that *S. calcitrans* repeatedly mate in a day could increase the probability of an infected individual to transmit conidia to several uninfected mates. This result is important as it demonstrates that ICIPE 30 could be used in an auto-dissemination control strategy to suppress the fly populations. In the field conditions, the incorporation of this fungal strain into trapping devices that massively catch and release *S. calcitrans* individuals would assist in spreading the fungus inoculum in the environment where the population density of the fly is high. The auto-dissemination control strategy has shown its effectiveness in other control system involving *Aedes albopictus* (Diptera: Culicidae) (Unlu et al., 2017) and this need to be validated under field condition in *S. calcitrans*.

We elucidated that *M. anisopliae* ICIPE 30 infection significantly impacted the feeding propensity of *S. calcitrans*. Our results showed that compared to uninfected controls, infected *S. calcitrans* took more time to consume blood. Also, these individuals were less likely to feed and when they did, they consumed a small amount of blood. For vector control purpose, reduction in feeding propensity is particularly important since pathogens they carry are transmitted during the blood meal. Pre-lethal reduction in feeding due to fungal infection has been shown in *An. stephensi* (Blanford et al., 2011, 2012), *Ae. aegyptis* (Darbro et al., 2012), and *An. gambiae* (Scholte et al., 2006). Studies indicate that this reduction in feeding is related to the fact that, individuals infected with fungi fail to locate potential blood sources owing to the reduction of the olfactory sensitivity occasioned by the infection. For example, George et al. (2011) showed that *B. bassiana* and *Metarhizium acridum* fungal spores, as well as inducing sublethal effects in *An. stephensi*, also reduce the responsiveness of its olfactory neurons. The reduction of feeding in infected *S. calcitrans* could also be due to the antifeedant activity of the secondary metabolites produced by the *M. anisopliae* blastopores, and the toxicity activity of these metabolites on the tissues of the insect midgut. For instance, Amiri et al. (1999) demonstrated that the destruxins produced by *M. anisopliae* have an antifeedant effect against larvae of *Plutella xylostella* (Lepidoptera: Plutellidae) and *Phaedon cochleariae* (Coleoptera: Chrysomelidae). Also, Skrobek and Butt (2005) showed that these molecules exhibit a cytotoxicity activity in *Spodoptera frugiperda* (Lepidoptera: Noctuidae) cells.

Our results also revealed that gravid females *S. calcitrans* differentiated substrates treated with *M. anisopliae* ICIPE 30 from substrates without this fungus. These females laid fewer eggs on treated substrates compared to the untreated ones (Figure 5Aii). Using *Metarhizium brunneum*, Machtinger et al. (2016) also found this fungus-induced oviposition avoidance in *S. calcitrans*. We showed that eggs laid on substrates with fungus had lower hatchability (Figure 5Aiii). This could explain why these substrates were avoided by gravid females *S. calcitrans*. In our previous studies, we showed that gravid females *S. calcitrans* were able to avoid substrates that could harm their progeny (Baleba et al., 2019, 2020). As the egg-laying decision of *S. calcitrans* is guided by olfactory cues (Baleba et al., 2019), we suggest that the avoidance of substrates treated with *M. anisopliae* by gravid females *S. calcitrans* could be mediated by chemical volatiles produced by *M. anisopliae*. Studies conducted on the termites *Macrotermes michaelseni* (Isoptera: Termitidae) (Mburu et al., 2011) and Coptotermes formosanus Shiraki (Isoptera: Rhinotermitidae) (Hussain et al., 2010) have already demonstrated the repellency of volatiles emitted by *M. anisopliae* conidia. Our results open new research avenues in identifying repellent odourant molecules from *M. anisopliae* that may be used to control *S. calcitrans* and reduce the spread of diseases that they transmit.

We found a reduction of eggs production in gravid females *S. calcitrans* infected with *M. anisopliae*. Also, we showed that the hatchability of eggs produced by these females was significantly low. These results suggest that once infected by *M. anisopliae, S. calcitrans* females are less likely to produce viable progeny for the next generation; contributing therefore to their population reduction. In *An. gambiae*, females infected with *M. anisopliae* laid fewer eggs (Scholte et al., 2006). Using *B. bassiana*, García-Munguía et al. (2011) also obtained a reduction of fecundity in infected females *Ae. aegytis*. The introduction of the entomopathogenic fungus, *Aspergillus parasiticus* (Eurotiales: Trichocomaceae) into natural populations of *An. gambiae, Culex fatigans*, and *Ae. aegypti* significantly reduced the fecundity and fertility of females that become infected (Nnakumusana, 1985). In our study, we hypothesize that the observed reduction of fecundity and fertility could be the immediate effect of the reduction of blood intake observed in *M. anisopliae* infected *S. calcitrans*. It has been shown that ovarian development, egg maturation and fertility heavily depend on the amount of blood taken by the insect (Gonzales and Hansen, 2016). Moreover, the reduction of fecundity and fertility in infected *S. calcitrans* could be associated with the effect of *M. anisopliae* on the maturation of *S. calcitrans* eggs. For example, Sánchez-Roblero et al. (2012) demonstrated that *B. bassiana* delay the maturation of *Anastrepha ludens* (Diptera: Tephritidae) eggs, resulting to the reduction the quantity of their mature basal oocytes and ultimately the number of eggs laid.

Also, we demonstrated that there is a fitness cost associated with the tolerance of *S. calcitrans* larvae to *M. anisopliae* infection. As Moraes et al. (2008), we found that *S. calcitrans* larvae were not susceptible to *M. anisopliae* infection. About 90% of the larvae contaminated with the 11 strains of *M. anisopliae* (Figure 6Ai) and those contaminated with high concentrations of ICIPE 30 (Figure 6Aii) reached the pupal stage. Moraes et al. (2014) demonstrated the existence of antifungal activity of *S. calcitrans* larvae against entomopathogenic fungal infection. The authors found that macerated solution of *S. calcitrans* larvae reduces the growth of *B. bassiana*; suggesting that these larvae produce robust anti-fungal substances protecting them from infection. When subjected to high-performance liquid chromatography (HPLC) analysis, new peaks in the chromatogram that could represent the antifungal molecules were obtained from the solution of macerated *B. bassiana* infected larvae as compared to the control group (Moraes et al., 2014). Earlier, a peptide affecting microorganism growth (stomoxyn) was identified in the exterior midgut of *S. calcitrans* (Boulanger et al., 2002). We recommend further studies to characterize molecules secreted by fungal infected *S. calcitrans* larvae and their protective role against pathogens.

Furthermore, we found that the tolerance of *S. calcitrans* larvae to *M. anisopliae* infection compromised some of their life-history traits. For instance, even though the majority of contaminated individuals from each larval instar pupated, their pupation period was longer than that of uncontaminated individuals. Also, these contaminated larvae were smaller, and in some contaminated larval instars such as the first and the third instars, adults had small weight and emergence percentage. Moreover, we obtained deformed adults from contaminated larvae even though their number was reduced. Vogelweith et al. (2017) explained that to optimize their response to pathogen invasion, individuals generally balance between investing in their immune system and other life-history traits. Thus, we argue that during a fungal infection in *S. calcitrans* larvae, certain fitness traits (e.g. developmental speed and weight gain) are suppressed in favor of immune reactions that will enhance the response against the infection.

In summary, our study examined the previously unexplored lethal and pre-lethal effect of *M. anisopliae* infection in *S. calcitrans*. We identified a potent and virulent *M. anisopliae* strain for *S. calcitrans* (ICIPE 30) that could be developed as biopesticide to manage the fly. We showed that the infectivity of this strain against *S. calcitrans* could be sex and age-dependent. Also, we proved that males and females of *S. calcitrans* contaminated with ICIPE 30 can horizontally transmit conidia of this fungal strain to their conspecific mates. Our results demonstrated that *M. anisopliae* ICIPE 30 infection reduces the feeding propensity, fecundity and fertility of *S. calcitrans* adults. We showed that the tolerance of *S. calcitrans* larvae to *M. anisopliae* infection has a fitness cost in these larvae. Taken together, our work provides detailed insights into the consequence of fungal infection of *S. calcitrans*, demonstrating the potentiality of the use of entomopathogenic fungi in controlling this important vector of various pathogens of human and veterinary significance. We recommend further chemical, molecular, and physiological studies that would additionally explain or elucidate our results.

## Data Availability Statement

The raw data supporting the conclusions of this article will be made available by the authors, without undue reservation.

## Author Contributions

SB: study conceptualization, experimental design, data collection, analysis and interpretation, and first manuscript draft preparation. AA: assistance in media preparation, fungus culture, germination test, and manuscript proofreading. MG and KA: advice in experimental design and manuscript proofreading. SS: manuscript proofreading. DM: study conceptualization, fund acquisition, advise in experimental design, supervision, and manuscript proofreading. All authors contributed to the article and approved the submitted version.

## Conflict of Interest

The authors declare that the research was conducted in the absence of any commercial or financial relationships that could be construed as a potential conflict of interest.

## Supplementary Material

Supplementary Figure 1 **(A)** Cylindrical plastic tube used as the contamination device. **(B)** Uninfected (i) and infected (ii) adults of *S. calcitrans* (Original Photo: Steve B.S. Baleba).

Supplementary Figure 2 Individual of *S. calcitrans* before **(A)**, during **(B)** and after the blood meal **(C)** (Original Photo: Steve B. S. Baleba).

Supplementary Figure 3 **(A)** Experimental setup where 10 gravid females were allowed to choose substrates with and without dried conidia of *M. anisopliae* ICIPE 30; **(B)** No choice oviposition bioassay setup using single uninfected (i) and infected (ii) female *S. calcitrans*.
