# Supplementary figures and images for: Infection of the Stable Fly, Stomoxys calcitrans, L. 1758 (Diptera: Muscidae) by the Entomopathogenic Fungi Metarhizium anisopliae (Hypocreales: Clavicipitaceae) Negatively Affects Its Survival, Feeding Propensity, Fecundity, Fertility, and Fitness Parameters
Source: Front Fungal Biol. 2021 Feb 24;2:637817. doi: 10.3389/ffunb.2021.637817 (PMC10512350; doi:10.3389/ffunb.2021.637817)

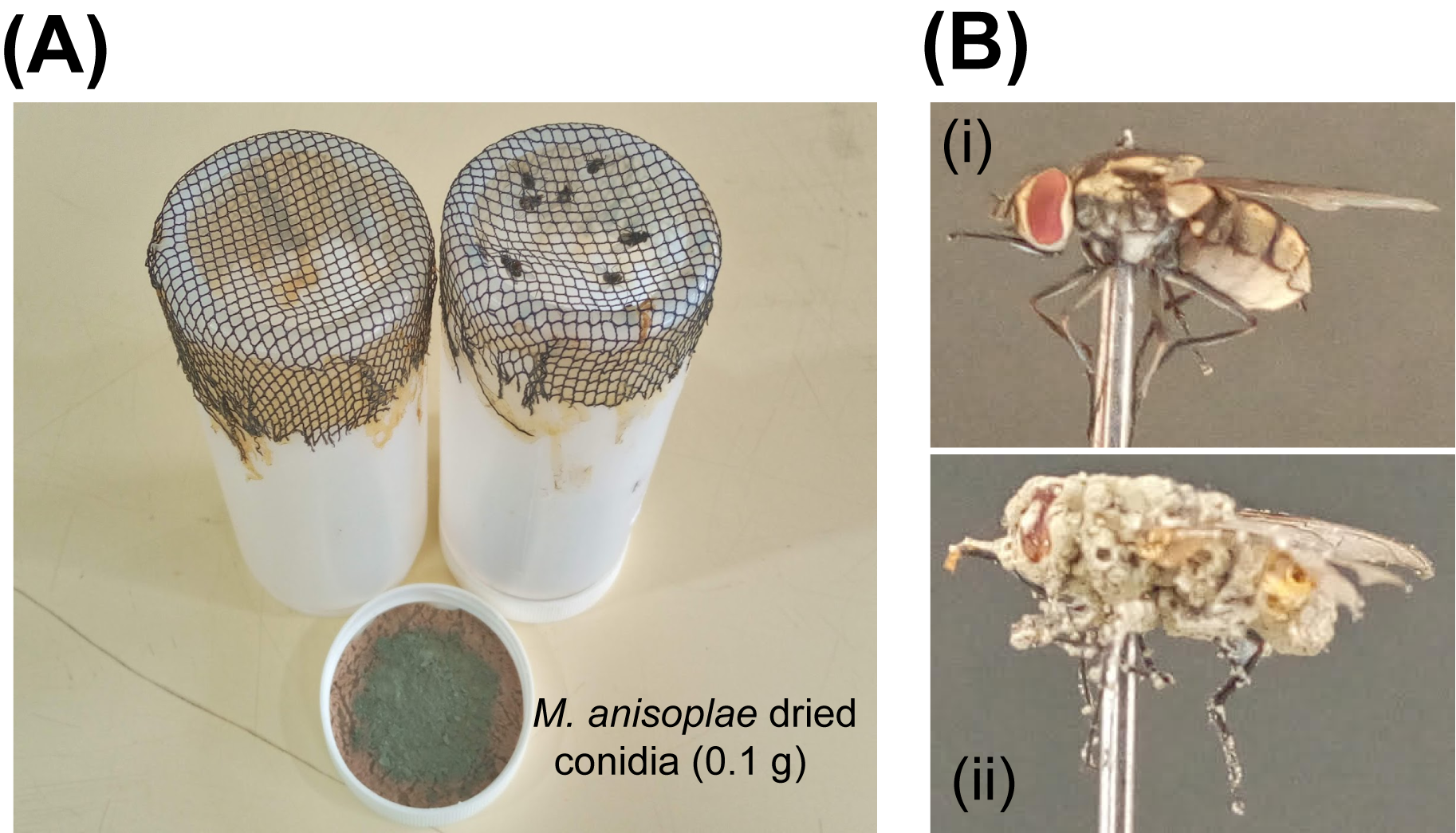

Supplement: Supplementary Figure 1 — (A) Cylindrical plastic tube used as the contamination device. (B) Uninfected (i) and infected (ii) adults of S. calcitrans (Original Photo: Steve B.S. Baleba). [file Image_1.TIF]

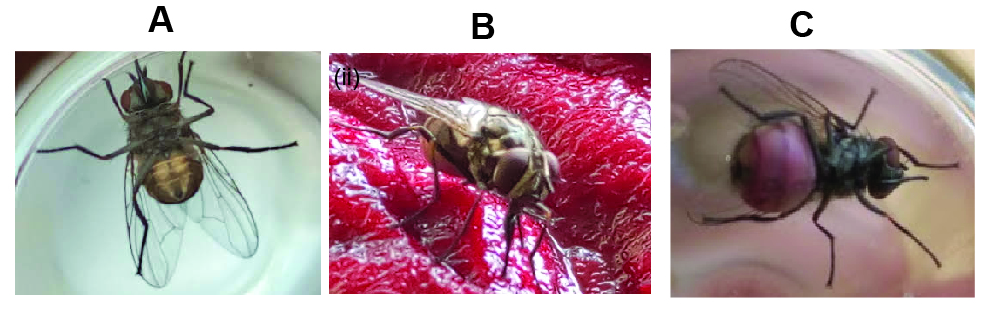

Supplement: Supplementary Figure 2 — Individual of S. calcitrans before (A), during (B) and after the blood meal (C) (Original Photo: Steve B. S. Baleba). [file Image_2.JPEG]

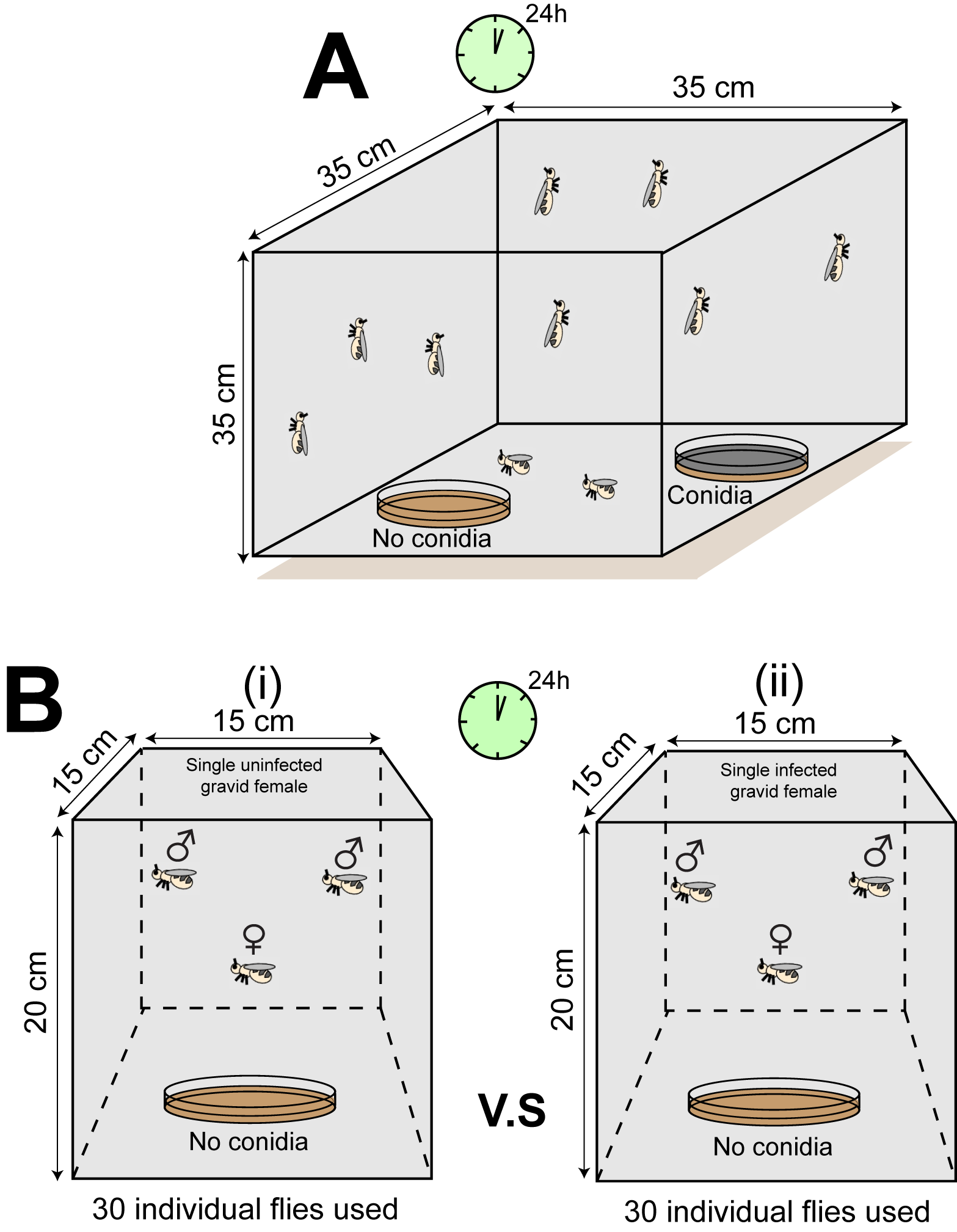

Supplement: Supplementary Figure 3 — (A) Experimental setup where 10 gravid females were allowed to choose substrates with and without dried conidia of M. anisopliae ICIPE 30; (B) No choice oviposition bioassay setup using single uninfected (i) and infected (ii) female S. calcitrans. [file Image_3.TIF]
